# Promises and challenges of personalized medicine to guide ARDS therapy

**DOI:** 10.1186/s13054-021-03822-z

**Published:** 2021-11-23

**Authors:** Katherine D. Wick, Daniel F. McAuley, Joseph E. Levitt, Jeremy R. Beitler, Djillali Annane, Elisabeth D. Riviello, Carolyn S. Calfee, Michael A. Matthay

**Affiliations:** 1 Cardiovascular Research Institute, University of California San Francisco, 513 Parnassus Avenue, HSE 760, San Francisco, CA 94143 USA; 2 Belfast Health and Social Care Trust, Royal Victoria Hospital and Wellcome-Wolfson Institute for Experimental Medicine, Queen’s University Belfast, Belfast, UK; 3 Division of Pulmonary, Allergy, and Critical Care Medicine, Stanford University, Stanford, CA USA; 4 Center for Acute Respiratory Failure and Division of Pulmonary, Allergy, and Critical Care Medicine, Columbia University, New York, NY USA; 5 Department of Intensive Care, FHU SEPSIS, and RHU RECORDS, Hôpital Raymond Poincaré (APHP), Garches, France; 6 Laboratory of Infection & Inflammation, School of Medicine Simone Veil, INSERM, University Versailles Saint Quentin, University Paris Saclay, Garches, France; 7 Harvard Medical School and Division of Pulmonary, Critical Care, and Sleep Medicine, Beth Israel Deaconess Medical Center, Boston, MA USA; 8 Departments of Medicine and Anesthesia, University of California, San Francisco, San Francisco, CA USA

**Keywords:** Acute respiratory distress syndrome, Acute lung injury, Personalized medicine, COVID-19, Clinical trials

## Abstract

Identifying new effective treatments for the acute respiratory distress syndrome (ARDS), including COVID-19 ARDS, remains a challenge. The field of ARDS investigation is moving increasingly toward innovative approaches such as the personalization of therapy to biological and clinical sub-phenotypes. Additionally, there is growing recognition of the importance of the global context to identify effective ARDS treatments. This review highlights emerging opportunities and continued challenges for personalizing therapy for ARDS, from identifying treatable traits to innovative clinical trial design and recognition of patient-level factors as the field of critical care investigation moves forward into the twenty-first century.

## Key lessons

- Personalized medicine in ARDS is inherently challenging because of heterogeneous etiology and pathophysiology
- ARDS research need not focus exclusively on novel investigational therapies, as repurposing drugs that have been studied in untargeted/unenriched populations could be just as innovative and promising, including for COVID-19 ARDS
- Opportunities for targeting therapies include timing, clinical phenotypes, and biologic phenotypes
- Adaptive clinical trial design offers the chance to investigate multiple therapies quickly and flexibly
- Supportive interventions, such as ventilator management and fluid strategy, can also potentially be personalized
- Though existing drugs and supportive care strategies may be repurposed/targeted, novel therapies are also on the horizon

## Introduction

**Fig. 1:**
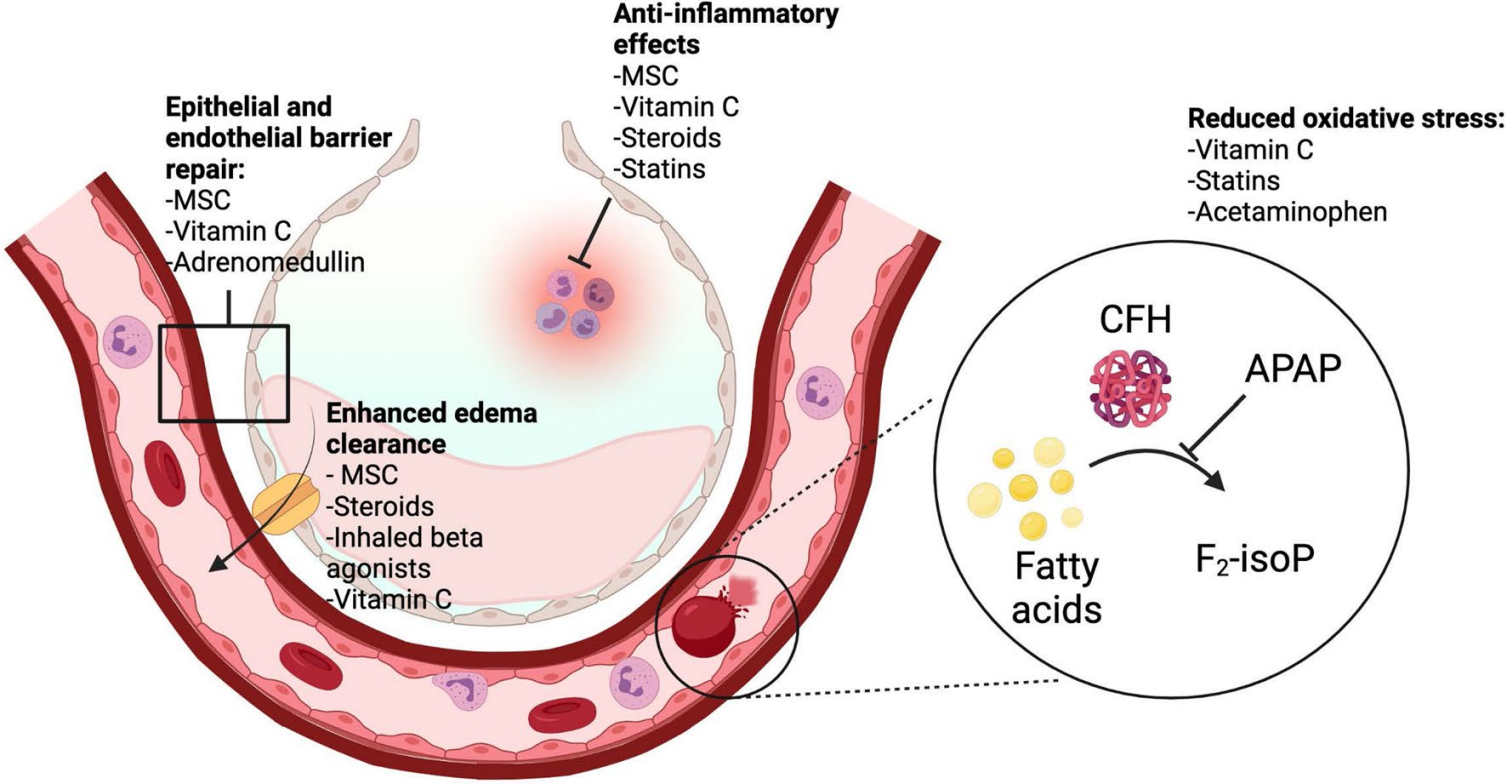
Biologic mechanisms in ARDS that may be targeted by various personalizable therapies. *MSC* mesenchymal stromal cells, *CFH* cell-free hemoglobin, *F*_*2*_*-IsoP* F_2_ isoprostanes, *APAP* acetaminophen. Figure created in BioRender

A challenge in personalizing therapy in critical illness syndromes including ARDS is their inherent heterogeneity. Perhaps in part because of this heterogeneity, years of investigation into possible therapies for classical ARDS have not confirmed the benefit of any pharmacologic treatment. Despite these challenges, the field of ARDS treatment remains rich for investigation. At least two biologic phenotypes of ARDS have been identified, first in secondary analyses of clinical trials [1, 2], and now in large observational cohorts [3]. These phenotypes appear to respond differentially to both investigational and standard supportive therapies [4, 5]. The understanding of not only the biology of ARDS, but also of its clinical presentation and timeline, is rapidly evolving [6]. Targeting both biologic phenotypes and specific clinical populations—for example, those that share a common risk factor or are identified early in their disease course—may be the key to advancing personalized medicine in ARDS. This review will consider specific pharmacologic and supportive therapies in ARDS that have not previously been proven to have benefit but that could hold promise if targeted to specific biological mechanisms (Fig. 1) or clinical/biologic phenotypes. Additionally, a new investigational therapy, mesenchymal stromal cells (MSCs), will be discussed. We will also explore the challenges of intelligent clinical trial design and the horizon for personalizing ARDS therapies in the global context.

### Targeting supportive therapy: personalized ventilator management

Several studies suggest that mechanical ventilation strategy might be personalized for improved outcomes in ARDS. Ventilation-induced lung injury (VILI) may occur in some patients even when the standard tidal volumes of 6 mL/kg predicted body weight (PBW) and plateau pressure ≤ of 30 cm H_2_O are targeted [7–10]. There is evidence that some patients benefit more than others from low tidal volume (LTV) and that the likelihood of benefit is associated with the baseline plasma concentration of the receptor for advanced glycation end products (RAGE), a biomarker of alveolar type I cell injury [11]. The recent REST trial demonstrated no benefit of extracorporeal carbon dioxide removal to facilitate ultra-LTV [12]. Whether baseline biologic characteristics could identify populations that may benefit is yet to be determined, though no pre-planned subgroup analyses showed evidence of heterogeneous treatment effect. Personalizing ventilator management entails assessing patient-specific risk of VILI and then weighing the potential risk/benefit of interventions intended to attenuate VILI (Table 1).

**Table 1:** Partial list of pivotal studies advancing precision ventilation for ARDS

Parameter and study	Key contribution
*Tidal volume*	
Hager et al. [92]	Reanalysis of the ARDSNet tidal volume trial demonstrated plateau pressure below 30 cm H_2_O was associated with additional improvement in survival, raising the possibility of residual VILI despite current standard-of-care low tidal volume ventilation
Amato et al. [13]	Reanalysis of several clinical trials demonstrated changes in airway driving pressure mediated effects of tidal volume (and PEEP) on mortality, suggesting driving pressure may be a useful metric for individualizing tidal volume to patient-specific mechanics
Pereira Romano et al. [19]	Pilot clinical trial demonstrated feasibility of a driving pressure-limited strategy without ECMO, laying the groundwork for a future trial of individualized tidal volumes
*PEEP*	
EPVent-1 and EPVent-2 trials [22, 93]	Single and multi-center trials, respectively, that demonstrated PEEP individualized to esophageal pressure, an estimate of pleural pressure, improved adjusted survival compared to an empirical low-PEEP strategy, but did not affect survival compared to an empirical high-PEEP strategy
Alveolar recruitment for ARDS trial (ART) [21]	Multicenter trial demonstrated a stepwise recruitment maneuver combined with PEEP titrated to highest respiratory system compliance, compared to an empirical low-PEEP strategy, increased mortality; interpretation of the PEEP effect is limited by the aggressive, prolonged exposure to extremely high airway pressures during the incremental/decremental recruitment maneuver
LIVE trial [23]	Multicenter trial demonstrated tailoring PEEP to radiographic findings (higher PEEP in patients with non-focal opacities, lower PEEP if focal opacities) did not improve survival compared to an empirical low-PEEP strategy, although misclassification of radiographs limits interpretation of findings
*Weighting relative importance of ventilator parameters*	
Gattinoni et al. [27]	Cohort study proposed mechanical power delivered by the ventilator, combining several ventilator parameters into a unifying metric to quantify VILI risk
Costa et al. [29]	Cohort study that concluded driving pressure and respiratory rate were the key parameters of mechanical power that influence mortality, also suggesting the effect on mortality of each 1 cm H_2_O increase in driving pressure was four times that of each 1 breath/min increase in respiratory rate

Several strategies have been proposed to individualize tidal volumes, including tailoring settings to airway driving pressure, end-inspiratory transpulmonary pressure, waveform-derived stress index, end-expiratory lung volume, and electrical impedance tomography [13–17]. Airway driving pressure (∆*P* = plateau pressure − PEEP) is perhaps the most readily accessible [13]. Adjusting tidal volume and PEEP to achieve driving pressures between 10 and 15 cm H_2_O has been proposed [18, 19], and is a reasonable though unproven target. A driving pressure-limited strategy in most patients not requiring extracorporeal membrane oxygenation (ECMO) is feasible in patients with ARDS [19], and refractory high driving pressure despite ventilator optimization may help select patients who would benefit from ECMO [20]. Clinical efficacy of a driving pressure-targeted strategy to individualize tidal volume and PEEP warrants prospective testing in clinical trials.

To date, no one strategy for individualizing positive-end expiratory pressure (PEEP) has proven superior for improving survival [21–25], perhaps in part because of the competing risk of overdistension with higher PEEP [26]. Personalized positive end-expiratory pressure (PEEP) titration should seek to minimize atelectrauma and driving pressure without exacerbating overdistension, but how best to achieve these objectives at the bedside remains unclear.

One challenge to personalized ventilator management is that there is no unifying marker of VILI or VILI risk. Higher risk is typically assumed in patients with more severe ARDS (lower PaO_2_/FiO_2_) or evidence of tidal overdistension. The energy delivered by the ventilator to the respiratory system per unit time, termed mechanical power, has been proposed to quantify VILI risk and guide ventilator titration [27]. Mechanical power correlates with VILI in animal models and mortality in clinical cohorts [28, 29]. However, mechanical power does not directly address atelectrauma or regional mechanical heterogeneity, and doubt exists regarding whether its empirical formulation correctly weighs importance of individual ventilator parameters. Further study is required to optimize identification of patients at risk of VILI and the approach for personalizing mechanical ventilation to mitigate this risk [29].

### Differential treatment responses in ARDS biologic phenotypes

Inflammatory phenotypes typically defined as hyperinflammatory/reactive and hypoinflammatory/uninflamed with distinct clinical outcomes have now been described in different settings in several studies of ARDS [1, 4, 30]. Recently, these inflammatory phenotypes have been described in both COVID-19 [31] and in patients with acute hypoxemic respiratory failure [32]. Thus, these phenotypes may represent “treatable traits” beyond the current syndromic definition of ARDS.

In a secondary analysis of the HARP-2 trial (simvastatin 80 mg or placebo in patients with ARDS), patients in the hyperinflammatory subgroup had significantly increased 28-day survival when randomized to simvastatin [4]. In contrast, in an analysis of the SAILS study (rosuvastatin 10 mg or placebo in patients with sepsis induced ARDS), there was no treatment effect in either group [2]. This difference may be related to differences in the study population (all-cause vs. sepsis-related) or the statins used (differences in dose and hydrophilicity).

Although these data support the concept of a precision medicine approach with simvastatin and other pharmacological therapies, it is important to highlight that these data should be regarded as hypothesis generating and need to be confirmed in prospective clinical trials. One final challenge to consider for enabling the translation of this precision medicine approach into prospective clinical trials and subsequently clinical practice is how to rapidly identify phenotypes at the bedside in real time. It is likely that plasma biomarkers, which are not routinely available, will be required to most accurately identify inflammatory phenotypes. The current lack of point-of-care assays represents a significant barrier to the clinical implementation of ARDS phenotypes. Ongoing clinical trials such as the PHIND trial (NCT04009330) aim to develop a point-of-care assay that can identify hyperinflammatory and hypoinflammatory phenotypes rapidly at the bedside. Such assays to facilitate identification of phenotypes to guide pharmacological therapy will be an important step in delivering precision medicine at the bedside.

### New avenues for existing therapies: sepsis-related ARDS

One strategy repurposing existing therapies in ARDS is to identify populations with shared clinical features or risk factors. For example, the role of cell-free hemoglobin (CFH) as a mediator of sepsis-related organ dysfunction is the basis of ongoing investigation in patients with ARDS due to sepsis. Although sepsis is itself a heterogeneous syndrome, the underlying pathobiology of patients with sepsis-related ARDS may differ from patients with ARDS from other causes, especially in the case of extrapulmonary sepsis [33, 34]. Targeting patients with sepsis for testing new therapies is a promising enrichment strategy for ARDS clinical trials. The effects of CFH on endothelial dysfunction, oxidative stress, and inflammation may have particular relevance to sepsis-related ARDS [35, 36].

The red blood cell membrane is altered in sepsis [37], leading to the release of free hemoglobin, a potent oxidizing and pro-inflammatory mediator [35]. Vitamin C and acetaminophen may diminish the injurious effects of CFH. Vitamin C infusion for ARDS was tested in the double blinded CITRIS-ALI trial. 167 patients with sepsis and ARDS were randomized to receive either 50 mg/kg vitamin C (*n* = 84) or placebo (*n* = 83) every 6 h for 96 h [38]. Patients in the vitamin C arm had significantly lower mortality (29.8% vs. 46.3% in the placebo group, *p* = 0.03), significantly more ICU-free days, and numerically more ventilator-free days [38].

Acetaminophen has also been shown to reduce concentrations of F_2_ isoprostanes, which are by-products of lipid peroxidation, among patients with sepsis [39, 40]. These promising findings for the benefit of acetaminophen and vitamin C in sepsis and sepsis-related ARDS are the basis for a planned phase 2 NHLBI-supported trial, Acetaminophen and Ascorbate in Sepsis: Targeted Therapy to Enhance Recovery (ASTER, NCT04291508), which will test both acetaminophen versus placebo and vitamin C versus placebo in a parallel design. Patients with sepsis and either evidence of shock or respiratory failure will be eligible for enrollment, facilitating an assessment not only of the effects of vitamin C and/or acetaminophen among patients with established ARDS due to sepsis, but also those at risk for sepsis-related ARDS. The ASTER trial is an important example of how a shared risk factor (sepsis) informs the investigation of repurposed therapies for ARDS.

### Lessons from the COVID-19 pandemic

**Fig. 2:**
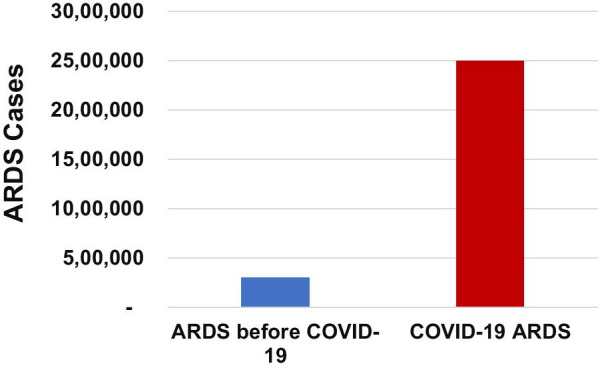
Estimated incidence of ARDS before and during the COVID-19 pandemic in the USA. ARDS prior to COVID-19 estimated from Rubenfeld et al., NEJM, 2005 (200,000/12 months or 300,000/18 months) [41]. COVID-19 ARDS estimated from 650,000 US deaths between March 2020 and September 2021, assuming 80% with severe pneumonia/ARDS and mortality of 20% from COVID-19 ARDS

A small benefit of the devastating COVID-19 pandemic is the opportunity for studying treatment effects in a large population of patients with viral pneumonia as a shared risk factor for ARDS. This unprecedented wave of respiratory viral infection has dramatically increased the incidence of ARDS. In the USA, for example, the estimated incidence of severe pneumonia/ARDS during the COVID-19 pandemic was approximately 2.5 million cases per 18 months, as compared to 300,000 cases per 18 months prior to the pandemic (Fig. 2) [41]. Treatments with uncertain benefit in undifferentiated ARDS, including corticosteroids and specific antagonists of the IL-6 receptor, probably prevent disease progression and death in hospitalized adults with COVID-19 (Table 2) [42–45], effects that are likely additive [42, 46]. Findings from these studies underscore the importance of the timing of interventions in the disease course of lung injury (discussed further below). In the RECOVERY trial, treatment with 6 mg of dexamethasone for 5 to 10 days resulted in a major survival benefit in patients with oxygen supplementation but not in those with early or mild disease who did not require oxygen support [47], suggesting a heterogeneous treatment effect by disease stage and severity. The survival benefit observed in RECOVERY trial participants receiving invasive mechanical ventilation (11% mortality reduction) was comparable to the benefits observed with 200 mg of hydrocortisone for 7 days in septic shock and ARDS (9% mortality reduction) [48].

**Table 2:** Summary of key studies on corticosteroids for non-Covid-19 and Covid-19-related ARDS

Studies	Date	Design	Participants	Interventions	Primary Outcome	Heterogeneity across subgroups	Comments
*Non-COVID-19 ARDS studies*							
Steinberg et al. [94]	2006	Country: USA Centers: 25 Placebo-controlled, double-blind 2 parallel groups	*N* = 180 (400 planned) Adults Persistent ARDS for 7 days at least and 28 days at most	Methylprednisolone bolus of 2 mg/kg followed by 0.5 mg/kg every 6 h for 14 days, 0.5 mg/kg every 12 h for 7 days, and then tapering of the dose Comparator: placebo	60-day mortality rates 29.2 (95% CI 20.8–39.4) versus 28.6 (20.8–38.6), *P* = 1.00	Methylprednisolone was associated with + 27% absolute difference in mortality rate in patients randomized after 14 days from onset of ARDS And + 26% in patients with low levels of procollagen peptide III in the bronchoalveolar lavage fluid	Sample size was changed from 400 to 180 owing to external information on baseline risk of death and low recruitment rate Methylprednisolone was associated with significantly more mechanical ventilation free days, and more risk of acquired muscle weakness
Meduri et al. [95]	2007	Country: USA Centers: 5 Placebo-controlled, double-blind 2 parallel groups Sequential analyses	*N* = 91 (400 planed) Adults ARDS within 72 h of onset	Methylprednisolone bolus of 1 mg/kg followed by 1 mg/kg/24 h continuous infusion for 14 days, 0.5 mg/kg/24 for 7 days, 0.25 mg/kg/day for 4 days, 0.125 mg/kg/day for 3 days Comparator: placebo 2:1 scheme	The RR for extubation of improvement in Lung Injury Score by 1 or more point at study day 7 was 1.96 (1.16–3.30) in favor of methylprednisolone	Interaction of responses to treatment and adrenal status by Synacthen test could not be performed owing to small sample size	36% of patients in the placebo group did received open label methylprednisolone
Tongyoo et al. [96]	2016	Country: Thailand Centers 1 Placebo-controlled, double-blind 2 parallel groups	*N* = 206 (194 planned) Adults Septic shock and ARDS	Hydrocortisone, 200 mg/day in 4 bolus of 50 mg for 7 days Comparator: placebo	The RR of dying at 28-day mortality was 0.82 (0.50–1.34) in favor of hydrocortisone	There was no interaction between response to treatment and subgroups based on age or severity of illness	Effects of corticosteroids were consistent across all secondary outcomes without increased in the risk of adverse reactions except for the risk of hyperglycemia
DEXA-ARDS [97]	2020	Country: Spain Centers: 17 Open-label 2 parallel groups	*N* = 277 (314 planned) Adults Moderate to severe ARDS	Dexamethasone 20 mg bolus daily to day 5, followed by 10 mg daily to day 10	The mean number of ventilator-free days was 4.8 days [95% CI 2.57 to 7.03] higher in dexamethasone group than in controls At 60-day the between-group difference in mortality was − 15.3%; − 25.9 to − 4.9 in favor of dexamethasone	–	Trial stopped prematurely for low recruitment rate Benefits from corticosteroids were consistent across secondary outcomes
*COVID-19 studies*							
RECOVERY [47]	2021	Country: UK Platform trial Multicenter, randomized Open-label	*N* = 6425 Adults Suspected or confirmed COVID-19 Hospitalized	Dexamethasone 6 mg/d orally or intravenously For 5 to 10 days Control: usual care	The age-adjusted rate ratio for 28-day mortality was 0.83; 95% CI 0.75–0.93	On invasive mechanical ventilation, the RR was, 0.64; 95% CI 0.51–0.81) On oxygen without invasive mechanical ventilation, the RR was 0.82; 95% CI 0.72–0.94 No respiratory support at randomization, the RR was 1.19; 95% CI 0.92–1.55	Trial stopped prematurely for efficacy
CoDEX [98]	2020	Country: Brazil Centers: 51 Open-label	*N* = 299 (350 planned) Adults Moderate to severe ARDS Onset < 48 h before randomization Invasive Mechanical ventilation Probable or confirmed COVID-19	Dexamethasone Intravenous bolus 20 mg/day for 5 days, then 10 mg/day for 5 days Control: usual care	The mean ventilator-free days was 6.6 (95% CI 5.0–8.2) in dexamethasone group versus 4.0 (95% CI 2.9–5.4) in controls The difference was 2.26; 95% CI 0.2–4.38, in favor of dexamethasone The RR for 28-day mortality was 0.86; 95% CI 0.64–1.15)	There were no evidence for interaction between response to treatment and age, severity of illness, degree of hypoxia, duration of disease prior to randomization, or vasopressor-dependency	Trial was stopped prematurely following external information from the RECOVERY trial
CAPE COVID [99]	2020	Country: France Centers: 28 Embedded, randomized Double-blinded Placebo controlled	*N* = 256 (290 planned) Adults Admitted to ICU or intermediate care unit Oxygen ≥ 6 L/min Probable or confirmed COVID-19	Hydrocortisone, continuous infusion for 8 days or 14 days 200 mg/day for 4 days or 7 days; 100 mg/day for 2 or 4 days, and 50 mg/day for 2 or 3 days Comparator: Placebo	The OR for 21-day mortality was 0.46 95% CI 0.20–1.04	–	Trial was stopped prematurely following external information from the RECOVERY trial
REMAP-CAP [100]	2020	Country: Europe, USA, Canada, Australia, New Zealand, Saudi Arabia Platform trial Centers, Platform trial Open-label Bayesian analyses	*N* = 403 ICU adults High-flow nasal oxygen with FIO_2_ ≥ 0.4 at ≥ 30 L/min, noninvasive or invasive Ventilatory support, or vasopressors Probable or confirmed COVID-19	Hydrocortisone intravenously fixed 7-day course of 50 mg or 100 mg every 6 h) OR A shock-dependent course of 50 mg every 6 h when shock was clinically evident) Comparator: usual care	The median adjusted OR and Bayesian probability of superiority were 1.43 (95% CI 0.91–2.27) and 93% for fixed-dose hydrocortisone, respectively, and 1.22 (95% CI 0.76–1.94) and 80% for shock-dependent hydrocortisone compared with no hydrocortisone	–	Trial was stopped prematurely following external information from the RECOVERY trial
MetCOVID [101]	2020	Country: Brazil Center, 1 Double-blinded Placebo 2 parallel groups	*N* = 416 (416 planned) ICU adults suspicion of COVID-19, SpO2 ≤ 94% with room air, required supplementary oxygen, or required IMV	Methylprednisolone intravenously 0.5 mg/kg twice daily for 5 days Comparator, Placebo	The OR for 28-day mortality was 0.92, 95% CI 0.67–1.28	There was no evidence for interaction between response to treatment and age, level of respiratory support, biomarkers of inflammation	Post hoc analysis suggested survival benefit from MP in patients of > 60 years old whereas younger patients may have increased risk of death with MP
GLUCOCOVID [102]	2021	Country: Spain Centers, 5 Open-label 2 parallel groups	*N* = 64 (180 planned) Adults Suspicion of COVID-19, disease duration < 7 days Moderate to severe ARDS CRP > 15 mg/L Or IL-6 > 20 pg/mL Or D-dimer > 800 ng/mL, or ferritin > 1000 mg/dL	Methylprednisolone intravenous 40 mg bid for 3 days, then 20 mg bid for 3 days Comparator: usual care	The age-adjusted RR for the composite of death, progression to ICU admission, or progression to NIV was 0.68, 95% CI 0.37–1.26, in favor of corticosteroids	There was no evidence for an interaction between treatment response and duration of symptoms prior to randomization	The study was stop prematurely following the release of the RECOVERY trial and the low recruitment rate

The variability in patients’ response to corticosteroids or IL-6 receptor antagonists may partly be explained by the variability in patients’ immune responses to SARS-CoV-2 [49], demonstrating that while investigating ARDS due to a common risk factor is an important strategy, it does not guarantee homogeneous response. The heterogeneity in COVID-19 patients’ responses to corticosteroids is in line with previous observations in non-COVID ARDS (Table 2) [50, 51]. Preventing the unsafe exposure of individuals to immunomodulating drugs such as corticosteroids and anti-cytokine monoclonal antibodies is of the utmost importance. Collectively artificial intelligence, omics tools and new generation of biomarkers may help designing individual fingerprints to guide immune modulation with corticosteroids in patients with ARDS regardless of the etiology or clinical phenotype (NCT04280497).

### Promising therapies in early ARDS: inhaled therapies

Targeting early ARDS may be another strategy for identifying personalizable therapies. For example, patients with early acute lung injury may be targeted to prevent progression. Inhaled delivery of therapies for early treatment of acute lung injury may provide the benefit of rapid delivery of therapeutic doses directly to the target organ with less systemic toxicity. Inhaled therapies are not strictly limited to early lung injury. There is an ongoing phase II trial of inhaled adrenomedullin for intubated patients with ARDS (NCT 04417036); however, how personalization may be relevant to this therapy has not yet been explored as its safety in all ARDS still needs to be established in this trial.

Inhaled beta agonists have been shown to achieve therapeutic levels in pulmonary edema fluid of patients with ARDS [52] and increase alveolar fluid clearance (AFC) in experimental models of lung injury [53]. In a phase II trial of patients with ARDS, systemic salbutamol decreased extravascular lung water [54]; however, subsequent phase III trials of systemic [55] and inhaled [56] beta agonists were stopped early for futility or signal for harm. Inhaled corticosteroids have been shown to reduce the severity of lung injury in experimental models of ARDS and have synergistic effects with beta agonists in the treatment of asthma and chronic obstructive lung disease. In a phase 2 a trial, Festic et al. showed improvement in oxygenation measured by categorical (± 20%) and continuous longitudinal change in the ratio of pulse oximetric oxygen saturation to the fraction of inspired oxygen (SpO_2_/FiO_2_) in 60 patients at risk for development of ARDS treated with a combination of aerosolized budesonide (0.5 mg/2 mL) and formoterol (20 mcg/2 mL) relative to placebo (4 mL 0.9% saline) [57]. Importantly, none of the 29 patients treated with aerosolized budesonide and formoterol had a > 20% deterioration in SpO_2_/FiO_2_. The strongest signal was in the largest subgroup of patients who had pneumonia as a risk factor for ARDS. Currently, these results are being tested in a randomized, placebo-controlled phase 2 trial of 600 patients hospitalized for severe pneumonia with hypoxemia. The primary endpoint is acute respiratory failure defined as any combination of HFNO, non-invasive ventilation (NIV), or IMV for > 36 h (ARREST PNEUMONIA, NCT04193878). Together, these studies demonstrate the potential benefit of repurposing inexpensive, safe medications for early use to prevent respiratory failure from various etiologies of acute lung injury.

An investigational agent, AP301, increases AFC by activating epithelial sodium channels (ENaC) in alveolar epithelial cells. In a phase 2 a trial of 40 mechanically ventilated patients with ARDS, Krenn et al. studied the effect of inhaled AP301 versus placebo (0.9% saline) [58]. Changes in extravascular lung water measured by PICCO^®^ thermodilution were not significantly different between groups overall, but extravascular lung water was significantly reduced in a post hoc subgroup of treated patients with greater baseline severity of illness (SOFA > 11). Early treatment of acute lung injury with inhaled therapies offers a promising potential paradigm shift for the prevention of respiratory failure. However, further study in phase 2 and 3 trials to identify optimal selection of agents, timing of delivery, and sub-phenotypes of patients to target is needed.

### Novel therapies: mesenchymal stromal cells

**Fig. 3:**
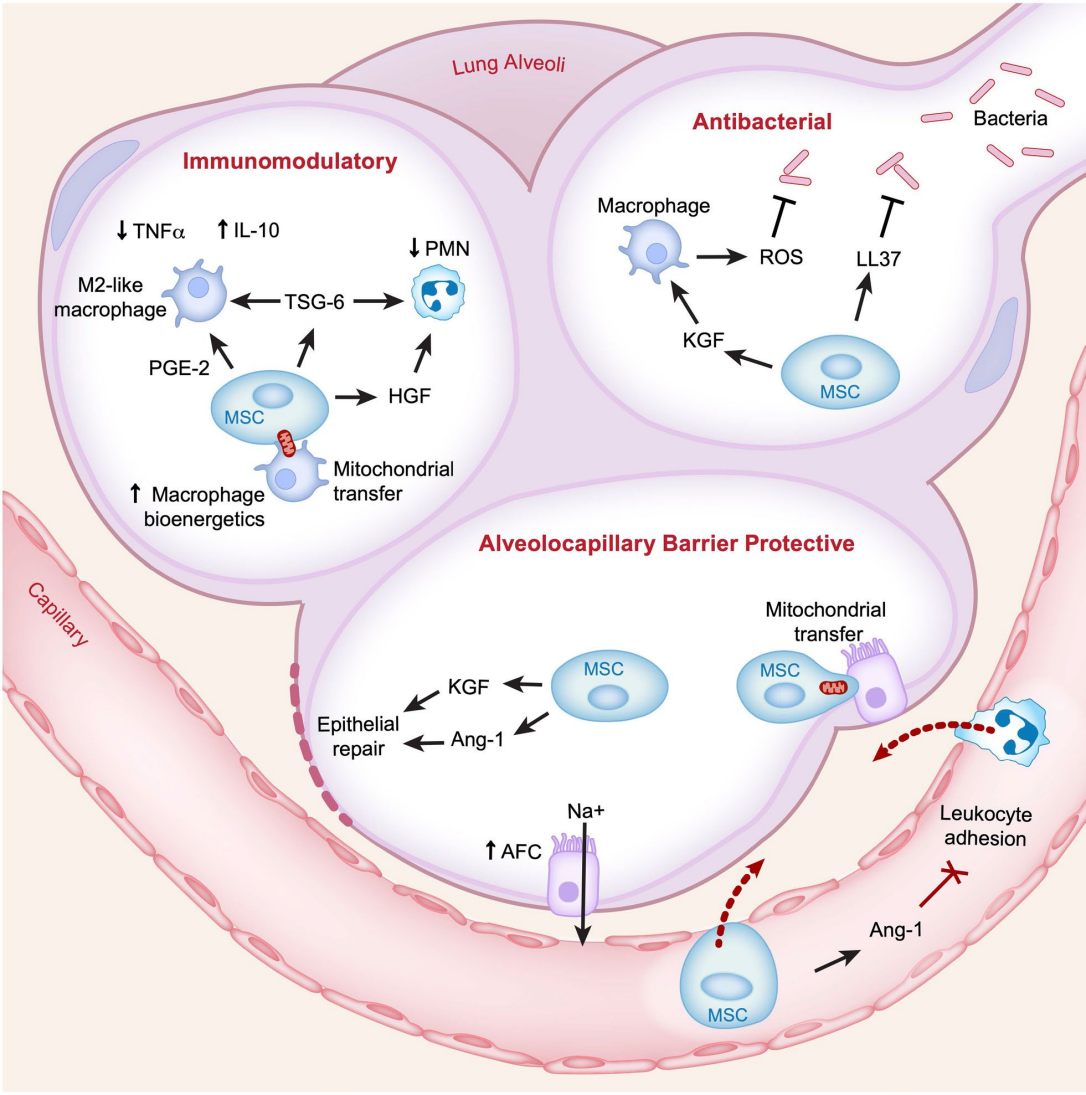
Mechanisms of MSC therapy for ARDS. *TNFα* tumor necrosis factor alpha, *IL* interleukin, *PMN* polymorphonuclear cells (neutrophils), *PGE-2* prostaglandin E2, *TSG-6* TNF stimulated gene 6, *HGF* hepatocyte growth factor, *ROS* reactive oxygen species, *LL37* cathelicidin antimicrobial peptide LL37, *KGF* keratinocyte growth factor, *Ang* angiopoietin, *AFC* alveolar fluid clearance

Personalization of novel therapies may rely on a detailed understanding of their biological mechanisms. There are considerable pre-clinical data that support the rationale for testing allogeneic mesenchymal stromal cells (MSCs) for the treatment of ARDS [59–61]. The potential mechanisms of benefit for reducing lung injury and enhancing lung repair are summarized in Fig. 3. MSCs can reduce lung vascular injury, perhaps in part by release of angiopoietin-1 that counteracts increased lung vascular protein permeability induced by angiopoietin-2 in both infectious and non-infectious causes of lung injury [62, 63]. In addition, MSCs may reduce the severity of epithelial injury by several pathways [64, 65]. MSCs also have anti-bacterial properties that have been demonstrated in both mice and the human lung, mediated by release of anti-microbial peptides such as LL-37 and an increase in macrophage phagocytosis [66].

Clinical trials have demonstrated no safety concerns and possible benefit in terms of improved oxygenation (by the oxygenation index) when analyzed by the viability of the MSCs [67, 68]. A recent study of the mini-BAL collected 48 h after treatment in a subset of these patients (27/60) showed that treatment with MSCs reduced BAL concentrations of total protein and mediators of lung injury, including sTNFR1 and angiopoietin-2 [69]. These data are the first direct evidence that MSCs can have a favorable effect on reducing the biologic severity of ARDS. A multicenter phase 2b trial of MSCs versus placebo for ARDS is underway (NCT03818854), and trials of MSCs for ARDS are in progress with considerable variation in the dose and source (bone marrow vs. umbilical cord for example) of the MSCs. Importantly, the biologic actions of MSCs in the airspaces likely depend upon the pulmonary microenvironment [70, 71], suggesting that populations may respond differentially to treatment depending upon their primary ARDS risk factor (for example, infectious etiology). Thus, there are likely further opportunities to personalize MSC therapy even if no benefit is observed in unselected populations.

### Intelligent clinical trial design

How should the heterogeneity of ARDS be incorporated in the approach to ARDS clinical trials? For some therapies, this heterogeneity may be irrelevant. The benefits of lung protective ventilation were demonstrated using a variation on the current clinical definition that captured patients with considerable variability in clinical severity, respiratory physiology, and biology [7, 72]. Supportive care trials might be relatively agnostic to underlying biologic pathways. The counter-argument to this more inclusive approach would be that heterogeneity of treatment effect has been identified even in trials of supportive care approaches [1, 5], including in the paradigmatic trial of low tidal volume ventilation [18], suggesting that clinical trials in ARDS should at least consider whether underlying heterogeneity is relevant. Table 3 provides an overview of incorporating heterogeneity into clinical trial design. The simplest approach is to pre-specify patient subgroups that will be analyzed for subgroup-specific benefits or harms. This approach has the advantage of not requiring a clear understanding of the optimal subgroup-therapy pairing/s and allowing for unexpected discoveries but is also inefficient and prone to type I error.

**Table 3:** Designing clinical trials to address heterogeneity within ARDS

Strategy	Type of heterogeneity	Specifics	Pros	Cons	Examples
Subgroup analysis	Any	Pre-specify subgroups for analysis on completion of traditional RCT	Acknowledges uncertainty about best matching of treatment to subgroup/phenotype	Inefficient; too many subgroups may result in false positives	Liu et al., activated protein C in ARDS [103]
Prognostic enrichment	Severity	Restrict enrollment to patients with more severe ARDS (lower PaO_2_/FiO_2_ ratio)	Likely enhances ability to detect treatment response, as relative risk reduction translates into higher absolute risk reduction if mortality is high	Reduces generalizability; may miss benefit in milder ARDS	PROSEVA trial [104]
Predictive enrichment	Biologic, physiologic, radiographic	Restrict enrollment to patients with specific abnormalities targeted by chosen therapy (e.g., inflammation of a certain level, for an anti-inflammatory therapy)	May identify treatment-responsive subset by better matching therapy with phenotype	Reduces generalizability; requires either understanding of or assumptions about best way to personalize treatment; no proof of “non-response” in excluded patients	RECOVERY tocilizumab trial [45]
Explicit comparison of personalized versus non-personalized therapy	Any	Randomize patients to personalized arm (with specific therapies based on subgroup/phenotype) vs standard-of-care arm	Explicit test of whether personalized strategy improves outcomes; tests effectiveness as well as efficacy to some degree	Complexity of design; misclassification may bias toward null; requires either understanding of or assumptions about best way to personalize treatment	LIVE trial [23]
Adaptive design	Any	Pre-specify subgroups and stratify randomization; adjust target population or randomization based on interim analyses of subgroup-specific results	Acknowledges uncertainty about best matching of treatment to subgroup/phenotype but with greater efficiency than standard RCT; allows “learning as you go”	Complexity of design; more sophisticated analytic approaches may be needed	Bhatt and Mehta (review) [105]

Two “enrichment” approaches to clinical trials may increase the chance of finding a therapeutic benefit: prognostic and predictive enrichment [73, 74]. Prognostic enrichment focuses on patients who are most likely to have poor disease-related outcomes. In ARDS, this approach typically means focusing on more severe disease. Predictive enrichment focuses on patients with a mechanistic phenotype most likely to be responsive to an intervention. During the COVID-19 pandemic, some trials of anti-inflammatory therapy have used this approach (e.g., RECOVERY’s focus on IL-6 blockade in patients with CRP > 75 mg/L) [45]. While both types of enrichment may increase the chance of identifying a signal, they also decrease generalizability and may risk missing effects in excluded patients. Predictive enrichment approaches informed by over-confidence in understanding of disease mechanisms could also potentially result in harm if the wrong patient subgroup is targeted [75]. Thus, predictive enrichment may be best reserved for situations in which a strong subgroup-specific treatment benefit has already been observed in more inclusive clinical trials [4].

One innovative approach to incorporating ARDS heterogeneity into clinical trials is to compare a personalized treatment strategy to a “one-size-fits-all” approach, as in the LIVE trial [23]. This study compared personalization of mechanical ventilation parameters to radiographic phenotype (diffuse vs. focal) to standard lung protective ventilation regardless of radiographic phenotype. This approach provides a measure of effectiveness in addition to efficacy, but the trial design is fairly complex and requires either an understanding of or assumptions about the best pairing between subgroup and treatment strategy.

A final innovative approach is the adaptive clinical trial. In its simplest form, this approach stratifies randomization by pre-specified subgroup and conducts interim analyses to identify subgroup-specific effects of treatment; one or more subgroups may then be dropped on the basis of these interim analyses. More complex iterations of this approach adjust randomization ratios to favor specific subgroups on the basis of interim results (so-called response-adaptive randomization), and/or incorporate a platform trial design facilitating multiple pairings of subgroups with treatments [76]. This approach allows trialists to learn as they go regarding the optimal pairing of treatment and subgroup while avoiding the inefficiencies of the standard RCT design with subgroup analysis only at trial conclusion. However, it also requires a much more complex statistical analytic approach that may be met with some skepticism by readers used to more traditional designs.

### A global perspective on personalized medicine for ARDS

Context, including regional and economic context, impacts the personalization of therapies for ARDS. Personalizing therapies to a specific clinical setting may be as necessary as individual patient personalization. While the overwhelming majority of critical care research occurs in high-income countries (HICs), 87% of the world’s population lives in low- or middle-income countries (LMICs) [77]. This economic context influences the predominant risk factors and biologic pathways leading to ARDS, the background physiologic environments of patients who develop ARDS, and the clinical resources available to diagnose and treat ARDS.

Different clinical insults predisposing to ARDS likely trigger different molecular pathways. While infection underlies ARDS in the majority of cases in HICs, trauma is a significant contributor in LMICs [78, 79]. Within infectious causes, malaria, dengue fever, and leptospirosis need to be studied in LMICs [80, 81]. One example of how this might be important, even for supportive therapies, is evident in sepsis care. While HIC studies have shown benefit from a clinical fluid resuscitation protocol for sepsis, a study of a comparable resuscitation protocol in Zambia demonstrated harm; one possible explanation for this finding was the high rate of subacute tuberculosis as the underlying cause of sepsis in the Zambian study [82]. Variability in patients’ background pathophysiology prior to the onset of ARDS may also have an impact on targetable molecular pathways. Patients in LMICs versus HICs have very different characteristics by age, nutritional status, body mass index, infectious versus non-communicable comorbidities, and potentially population genetic trends. All of these may influence the targets for ARDS therapy in a given patient or population.

Resources may also impact which therapies provide benefit. For example, high flow nasal oxygen (HFNO) has been shown to decrease intubation rates but has not consistently reduced mortality in HICs [83]. In settings where intubation and mechanical ventilation are frequently unavailable, a decrease in need for intubation could conceivably translate to improvement in mortality. One study found that HFNC could decrease mortality in a model simulating scarce ventilators secondary to COVID-19 [84]; another study in children with hypoxemia in East Africa suggests that HFNC may confer a mortality benefit in that setting [85].

For personalized medicine in ARDS to be globally relevant, it needs to be developed in all regions of the world, including the resource-variable settings of LMICs [86]. This means that ARDS must be defined in such a way that it can be recognized and studied in a wide variety of resource contexts [79, 87]. This also requires a commitment to invest in the staff and infrastructure needed for diverse LMIC sites across the world to participate in ARDS trials [88]. The details of epidemiologic, resource, and practice characteristics must be documented, so that differences in study outcomes between sites can be understood and interpreted within the contexts in which they were produced [86]. Finally, studies in HIC sites should include both more and less complex diagnostics to allow correlations to be made and validated, thus facilitating the use of less complex diagnostics in LMICs. This includes imaging (chest radiograph vs. ultrasound), oxygenation (arterial blood gases vs. pulse oximetry), and biomarkers (plasma biomarkers vs. readily available clinical data) [89–91]. Personalizing ARDS treatments within discrete HIC populations and hoping that these therapies will translate to the majority of the world living in LMICs is not an adequate strategy. The development of targeted therapies for ARDS must include diverse peoples and populations from the outset.

## Conclusions

The mainstay of ARDS treatment remains optimal supportive therapy with lung protective ventilation, proning, and a fluid conservative strategy, but the prospect of personalized therapies offers promise for further advances in treating ARDS. Although the heterogeneity of ARDS in some ways presents a challenge for personalization, it also provides a rich landscape with many opportunities for further investigation. By identifying clinical and biological characteristics that may differentially respond to existing and investigational treatments, clinical trials can be enriched in an adaptive manner. Pharmacologic and supportive interventions can be targeted by the stage of the syndrome of respiratory failure (such as early vs. late ARDS), ARDS risk factor, emerging biologic phenotypes, and individual pulmonary mechanics. These investigations must take into account variable resources as the study of ARDS and other critical illness syndromes expands globally to ensure that new discoveries carry maximal impact across diverse populations.

## Data Availability

Data sharing not applicable to this article as no datasets were generated or analyzed during the current study.
